# Machine learning reveals dynamic controls of soil nitrous oxide emissions from diverse long‐term cropping systems

**DOI:** 10.1002/jeq2.20637

**Published:** 2024-10-09

**Authors:** Jashanjeet Kaur Dhaliwal, Dinesh Panday, G. Philip Robertson, Debasish Saha

**Affiliations:** ^1^ Biosystems Engineering and Soil Science University of Tennessee Knoxville Tennessee USA; ^2^ Rodale Institute Kutztown Pennsylvania USA; ^3^ W. K. Kellogg Biological Station Michigan State University Hickory Corners Michigan USA; ^4^ Department of Plant, Soil, and Microbial Sciences Michigan State University East Lansing Michigan USA

## Abstract

Soil nitrous oxide (N_2_O) emissions exhibit high variability in intensively managed cropping systems, which challenges our ability to understand their complex interactions with controlling factors. We leveraged 17 years (2003–2019) of measurements at the Kellogg Biological Station Long‐Term Ecological Research (LTER)/Long‐Term Agroecosystem Research (LTAR) site to better understand the controls of N_2_O emissions in four corn–soybean–winter wheat rotations employing conventional, no‐till, reduced input, and biologically based/organic inputs. We used a random forest machine learning model to predict daily N_2_O fluxes, trained separately for each system with 70% of observations, using variables such as crop species, daily air temperature, cumulative 2‐day precipitation, water‐filled pore space, and soil nitrate and ammonium concentrations. The model explained 29%–42% of daily N_2_O flux variability in the test data, with greater predictability for the corn phase in each system. The long‐term rotations showed different controlling factors and threshold conditions influencing N_2_O emissions. In the conventional system, the model identified ammonium (>15 kg N ha^−1^) and daily air temperature (>23°C) as the most influential variables; in the no‐till system, climate variables such as precipitation and air temperature were important variables. In low‐input and organic systems, where red clover (*Trifolium repens* L.; before corn) and cereal rye (*Secale cereale* L.; before soybean) cover crops were integrated, nitrate was the predominant predictor of N_2_O emissions, followed by precipitation and air temperature. In low‐input and biologically based systems, red clover residues increased soil nitrogen availability to influence N_2_O emissions. Long‐term data facilitated machine learning for predicting N_2_O emissions in response to differential controls and threshold responses to management, environmental, and biogeochemical drivers.

## Core Ideas

- Long‐term (2003−2019) data from an LTAR/LTER site was used to understand dynamic controls of N_2_O emissions.
- The RF model showed different drivers and threshold conditions influencing N_2_O emissions in long‐term rotations.
- Soil ammonium and daily temperature were identified as the most influential variables in the Conventional system.
- Climate variables—precipitation and daily temperature—were important variables in the No‐till system.
- In low‐input and organic systems, nitrate from legume cover crops strongly influenced N_2_O emissions.

**Abbreviations**
CT: conventionally tilled
C–S–W: corn–soybean–winter wheat
GWC: gravimetric water content
NT: no‐till
OOB: out‐of‐bag
PD: partial dependence
RF: random forest
RMSE: root mean square error
UAN: urea ammonium nitrate
WFPS: water‐filled pore space

## 1 INTRODUCTION

Global terrestrial emissions of nitrous oxide (N_2_O), a potent greenhouse gas, have increased from 6.3 Tg N_2_O‐N year^−1^ in the preindustrial era to 10 Tg N_2_O‐N year^−1^ in the last decade (2007–2016), which represents an increase in atmospheric N_2_O load of ∼60% (Tian et al., 2019). This increase is largely governed by the increase in cropland soil emissions from 0.3 to 3.3 Tg N_2_O‐N year^−1^ during the same period. Increases in fertilizer nitrogen (N) application rates drive N_2_O emissions from agricultural soils—the largest anthropogenic source of atmospheric N_2_O that contributes nearly 10% of global anthropogenic radiative forcing (Butterbach‐Bahl et al., 2013; Shcherbak et al., 2014; Syakila & Kroeze, 2011; Zhang et al., 2020).

Nitrous oxide emissions from agricultural soils result from biogeochemical processes primarily dominated by microbial nitrification and denitrification (Dobbie & Smith, 2003; Robertson & Groffman, 2024). These processes are highly sensitive to environmental conditions including soil moisture/water‐filled pore space (WFPS), temperature, pH, and availability of organic carbon (C) and inorganic N (Baral et al., 2022; Giltrap et al., 2010; Oertel et al., 2016)—further influenced by management practices. Net N_2_O emissions measured in situ are the outcomes of complex interactions among the driving factors and their threshold conditions that are often nonlinear and spatially discontinuous (Robertson, 2023). For this reason, the prediction of N_2_O emissions based on simultaneously observed environmental factors and N substrate concentrations shows very weak to no correlations in most studies (e.g., Gelfand et al., 2016; Maharjan & Venterea, 2013; Wanyama et al., 2018). This complexity is often manifested in the highly dynamic and variable nature of soil N_2_O emissions characterized as “hot spots” and “hot moments” (Groffman et al., 2009; Saha et al., 2018; Venterea et al., 2012). Only a fraction of this spatial and temporal variability may be attributed to applied fertilizer N, with much of the remainder attributed to the soil, climatic, and management factors influencing total N_2_O emissions (de Klein et al., 2020; Deng et al., 2022; Groffman et al., 2009). Both direct observations and meta‐analyses show a non‐linear relationship between N fertilization rate and N_2_O emissions (e.g., Hoben et al., 2011; Kim et al., 2013; Millar et al., 2018; Scheer et al., 2016; Shcherbak et al., 2014), which further highlights the confounding impacts of multiple driving factors on N_2_O emissions.

Long‐term management practices such as no‐till (NT) and cover cropping alter soil biophysical and biogeochemical conditions to modify the shape and threshold response of N_2_O emissions to environmental and biogeochemical drivers. For example, long‐term NT improves soil physical properties such as soil aeration and moisture retention and reduces soil temperature (Nouri et al., 2019). While lower soil temperature and improved aeration under macropore‐dominated NT soils may reduce N_2_O emissions (Ussiri et al., 2009; Van Kessel et al., 2013), moist soils can promote N_2_O emissions from denitrification and its rapid escape to the atmosphere due to greater diffusivity (Wang & Zou, 2020). Therefore, N_2_O response to soil moisture and temperature may differ between NT and conventionally tilled (CT) soils, which makes it difficult to predict management impacts on N_2_O emissions under inter‐annual weather variability. Literature inconsistently shows that N_2_O emissions under NT either decrease (Grandy et al., 2006; Six et al., 2004; Van Kessel et al., 2013) or increase (Ball et al., 2008; Mei et al., 2018) compared to CT. Similarly, the quantity and quality of cover crop biomass influence soil N availability, which in turn affects N_2_O emissions and their response to fertilizer N application (Finney et al., 2015; Panday et al., 2022). Following cover crop termination, legume residues can significantly increase N_2_O emissions due to fast N release (Davis et al., 2019; Saha, Kaye, et al., 2021). Accelerated cover crop decomposition and heterotrophic respiration rapidly consume soil oxygen (O_2_) to promote anoxia and large N_2_O emissions from denitrification regardless of soil moisture conditions (Lussich et al., 2024). These findings are in contrast with the generalized conclusions about negligible impacts of cover crops on soil N_2_O emissions (Basche et al., 2014; Kaye & Quemada, 2017). Such inconsistencies further highlight our limited understanding of dynamic variable controls on N_2_O emissions in response to environmental and management differences.

**Core Ideas**

- Long‐term (2003−2019) data from an LTAR/LTER site was used to understand dynamic controls of N_2_O emissions.
- The RF model showed different drivers and threshold conditions influencing N_2_O emissions in long‐term rotations.
- Soil ammonium and daily temperature were identified as the most influential variables in the Conventional system.
- Climate variables—precipitation and daily temperature—were important variables in the No‐till system.
- In low‐input and organic systems, nitrate from legume cover crops strongly influenced N_2_O emissions.

Additionally, long‐term N_2_O emissions are greatly influenced by inter‐annual variability in weather conditions (Baral et al., 2022; Burchill et al., 2014), and in particular variability in rainfall distributions (Rowlings et al., 2015). However, many studies investigating spatial and temporal N_2_O emission controls are based on short‐term measurements spanning from one to two growing seasons and capturing only a snapshot within a growing season (Dorich et al., 2020). The time‐consuming and resource‐intensive nature of chamber‐based N_2_O flux measurements is a key limitation for individual research projects in implementing spatially and temporally extensive flux monitoring. Coordinated efforts by long‐term research network sites can be useful in overcoming such limitations by providing multi‐year data capturing weather variability and management legacies (e.g., crop rotation, NT, and cover cropping), which often take time to emerge (Cusser et al., 2020; Six et al., 2004).

Several quantitative tools have been traditionally employed to understand the complexity of soil, climate, and crop management practices influencing N_2_O emissions with varying degrees of success. For example, parametric regression models, by design, do not represent nonlinear variable interactions influencing N_2_O emissions (Kim et al., 2013). Similarly, the emission factor (EF) approach by the Intergovernmental Panel on Climate Change (IPCC) is insensitive to dynamic interactions between the environmental factors and management conditions. These models often fail to predict how N_2_O emissions may change at a finer temporal and spatial scale (Ramírez‐Melgarejo et al., 2020; Richards et al., 2016). Furthermore, these approaches are limited to provide insights on critical values of predictor variables differentially influencing N_2_O under different management practices, such as crop diversification, NT, cover crop, and so forth. Unlike the empirical models, process‐based biogeochemical models can simulate feedback and interactions that can be difficult to distinguish in the field (Giltrap et al., 2010). Process‐based models, such as the DayCent (Del Grosso et al., 2000; Parton et al., 2001) and DeNitrification‐DeComposition (Li, 2000, 2007), have considered important regulating factors to support the prediction of N_2_O emissions and thus have been recognized as useful tools to evaluate the effects of management practices on N_2_O emissions from agricultural soils (Deng et al., 2018; Jarecki et al., 2008). However, heavy parametrization and site‐specific calibration need of the process‐based models often limit their extensive use (Ehrhardt et al., 2018; Fuchs et al., 2020; Gaillard et al., 2018; Gilhespy et al., 2014).

Machine learning models such as decision trees and random forest (RF) treat the output variable (e.g., N_2_O) as an implicit function of input features (e.g., soil, environment, and management), and can capture complex nonlinear relationships as learned from the data and not by predefined process‐based relationships as in the case of biogeochemical models (Breiman, 2001; Huang et al., 2010). The RF appears to be a more promising technique than classical regression‐based methods and other machine learning algorithms due to its ability to rank predictors using internal measures of variable importance and to provide valuable insights through partial dependence (PD) plots (Djiemon et al., 2019; Saha et al., 2017).

Machine learning has been increasingly used to predict N₂O emissions in recent years (Glenn et al., 2021; Joshi et al., 2024; Liao et al., 2023; Philibert et al., 2013; Saha, Basso, & Robertson, 2021; Yin et al., 2022). However, predictive ability of machine learning models is correlational as learned from the data, hence has limited power in representing a novel scenario, which is a key pursuit of process‐based models. Nonetheless, machine learning models can be resource efficient in scaling our existing knowledge and provide insights on key variable interactions controlling N_2_O emissions to optimize process‐based models. The availability of long‐term data creates novel opportunities for using machine learning models to understand differential controls of N_2_O emissions under diverse management practices.

By leveraging 17 years of long‐term observations, we used RF and decision tree models to infer the controls and drivers of soil N_2_O emissions from four corn (*Zea mays* L.)–soybean (*Glycine max* L.)–wheat (*Triticum aestivum* L.) rotations employing diverse tillage, fertilization, and cover cropping practices in the US upper Midwest. Our objectives are to identify critical management, environmental, and biogeochemical drivers of N_2_O emissions and their differential relationships and threshold conditions for emissions under diverse long‐term cropping rotations.

## 2 MATERIALS AND METHODS

### 2.1 Site description

Our study tracks a 17‐year (2003–2019) long‐term data stream from the Main Cropping System Experiment (MCSE) of the Kellogg Biological Station (KBS) Long‐Term Ecological Research (LTER) site, which is also one of 18 Long‐Term Agroecosystem Research sites across the United States. Historical data on yearly N_2_O emissions, soil properties, agricultural management practices, and weather were obtained from the KBS LTER data catalog (https://lter.kbs.msu.edu/datatables). The KBS LTER site is located in the northeast portion of the US corn belt in southwest Michigan (42°24′ N, 85°24′ W, and 288 masl) and was originally established in 1987 to examine the ecology of intensively managed field crops and the landscape in which they reside (Robertson & Hamilton, 2015). Soils at the site are well‐drained Typic Hapludalfs of the Kalamazoo (fine‐loamy, mixed, mesic) and Oshtemo (coarse‐loamy, mixed, mesic) series, formed from glacial till and outwash with some intermixed loess (Crum & Collins, 1995; Luehmann et al., 2016). Surface soils exhibit an average of 43% sand and 17% clay contents (Robertson & Hamilton, 2015), with an average organic C of 11.9 g kg^−1^, total N of 1.2 g kg^−1^, and pH of 6.5. The local weather is humid continental with hot and wet summers. Annual air temperature (30‐year average) at KBS is 9.9°C, and precipitation averages 1027 mm year^−1^ evenly distributed seasonally with a snowfall of about 1.4 m and an average snow depth of 148 mm for days when snow is present (Robertson & Hamilton, 2015). Details about weather conditions during the crop‐growing season period are given in Figure S1.

### 2.2 Cropping systems and management

The MCSE includes seven treatments arranged in a randomized complete block design with six replications; we used four annual crop treatments (Table 1), including: (1) a conventional system with chisel tillage and standard chemical inputs, (2) an NT system with standard chemical inputs, (3) a reduced input system with chisel tillage, low fertilizer inputs, and cover crops, and (4) a biologically based/organic system managed organically using chisel tillage, cover crops, and no synthetic chemical inputs. The NT system was identical to the conventional system except for the lack of tillage. Chisel tillage in conventional, reduced input, and biologically based/organic systems was conducted to a depth of 15–18 cm, followed by secondary tillage operations such as disking. Since 1993, all of the systems have been maintained as corn–soybean–winter wheat (C–S–W) rotations according to best management practices (Robertson, 2015; Robertson & Hamilton, 2015). Corn and soybean were planted in late April or May and winter wheat was planted in late September or early October. The conventional and NT systems received recommended rates of N fertilizer at 137 ± 20 kg N ha^−1^ year^−1^ during the corn phase and 77 ± 17 kg N ha^−1^ year^−1^ during the wheat phase of each rotation (Gelfand et al., 2016). Corn was managed with split fertilizer application ∼30 kg N ha^−1^ at planting, with the remainder side‐dressed at the V6 stage around June 28 (±9 days), while wheat was fertilized on April 19 (±7 days). The reduced input system received N fertilizer at an average rate of 30 ± 3 kg N ha^−1^ year^−1^ on May 13 (±7 days) during the corn phase and 40 ± 13 kg N ha^−1^ year^−1^ in April during the wheat phase of the rotation. No N fertilizer was applied to the soybean phase, but it received minor N fertilizer inputs as part of phosphorus (P), potassium (K), and herbicide applications, which were applied as needed in some years according to the Michigan State University recommendations (Warncke et al., 2009). The biologically based/organic system is a USDA‐certified organic treatment. No manure, compost, or insecticide was applied in any of the cropping systems. Nitrogen fertilizer was added as urea ammonium nitrate (UAN) injected at 10‐cm depth between crop rows at planting and as side‐dressing. Soybean and corn were harvested in October and November, respectively, and winter wheat was harvested in July. In the low‐input and biologically based/organic systems, the winter cover crop cereal rye (*Secale cereale* L.) was planted in October following corn and before soybean, and red clover (*Trifolium pratense* L.) was frost‐seeded into winter wheat in March and terminated just before planting corn the following spring.

**TABLE 1:** Agronomic management practices under the four annual cropping systems studied (2003–2019).

Cropping system	Crop phase (N fertilizer, kg N ha^−1^)				
Conventional	Corn (137 ± 20)		Soybean	Wheat (77 ± 17)	
No‐till	Corn (137 ± 20)		Soybean	Wheat (77 ± 17)	
Reduced input	Corn (30 ± 3)	Cereal rye	Soybean	Wheat (40 ± 13)	Red clover
Biologically based/organic	Corn	Cereal rye	Soybean	Wheat	Red clover

Plots in conventional, reduced input, and biologically based/organic were chisel plowed, while those in NT were not tilled. Cereal rye was sown following corn before soybean and red cover was frost‐seeded into winter wheat in reduced input and biologically based/organic cropping systems.

### 2.3 Data collection

Nitrous oxide flux measurements were made in four out of the six replicates in each treatment using static chambers at weekly to monthly intervals when the soils were not frozen. The manual open‐bottom chambers, equipped with rubber septa and measuring 29 cm × 29 cm × 14 cm, were made from opaque polycarbonate sheeting and placed on semipermanent aluminum bases (28 cm × 28 cm × 10 cm), which were removed only during agronomic activities. Each chamber had a volume of approximately 12 L. Gas samples were collected using a headspace‐flushed syringe every 15 min over a 1‐h chamber closure period. The samples were stored in 5.9‐mL Exetainer vials (Labco Limited) and analyzed using gas chromatography. The gas flux was calculated using the equation:

$$F=\frac{a*M*P*V}{A*R*T}$$

where *F* is gas flux (g N cm^−2^ h^−1^), *a* is the average rate of change of gas concentration (ppm h^−1^), *M* is molecular weight of N in N_2_O (28 µg N µmol N_2_O^−1^), *P* is assumed atmospheric pressure (1 atm), *R* is universal gas constant (0.0821 L‐atm mol‐K^−1^), *T* is field temperature (°K = °C + 273), *V* is volume of gas in chamber (cm^3^), and *A* is soil surface area covered by chamber (cm^2^).

More details on N_2_O flux measurements can be found in Gelfand et al. (2016) and at https://lter.kbs.msu.edu/protocols/113. Long‐term gas sampling frequency was designed to capture treatment differences. During each gas sampling event, soil cores from the 0‐ to 25‐cm depth were extracted from near the chamber for determination of gravimetric water content (GWC). WFPS was determined using the GWC and a consistent bulk density of 1.44 g cm^−3^ across all treatments. Soil samples (0‐ to 25‐cm depth) from five random locations in each plot were collected biweekly each year for inorganic N (${\mathrm{NH}}_{4}^{+}$ and ${\mathrm{NO}}_{3}^{-}$) concentrations. Soils were extracted with 2 M KCl and analyzed for ${\mathrm{NH}}_{4}^{+}$ and ${\mathrm{NO}}_{3}^{-}$ concentrations in a continuous flow analyzer (Alpkem 3550; O.I. Analytical). Soil ${\mathrm{NH}}_{4}^{+}$, ${\mathrm{NO}}_{3}^{-}$, and WFPS values were linearly interpolated between two sampling dates to match the N_2_O sampling dates when soil samples were not collected on the gas sampling days due to management and weather reasons. Cumulative gas fluxes were calculated by linear interpolation between the successive sampling dates. The 17‐year database includes five full W–C–S rotations under each treatment.

### 2.4 Statistical methods

**FIGURE 1:**
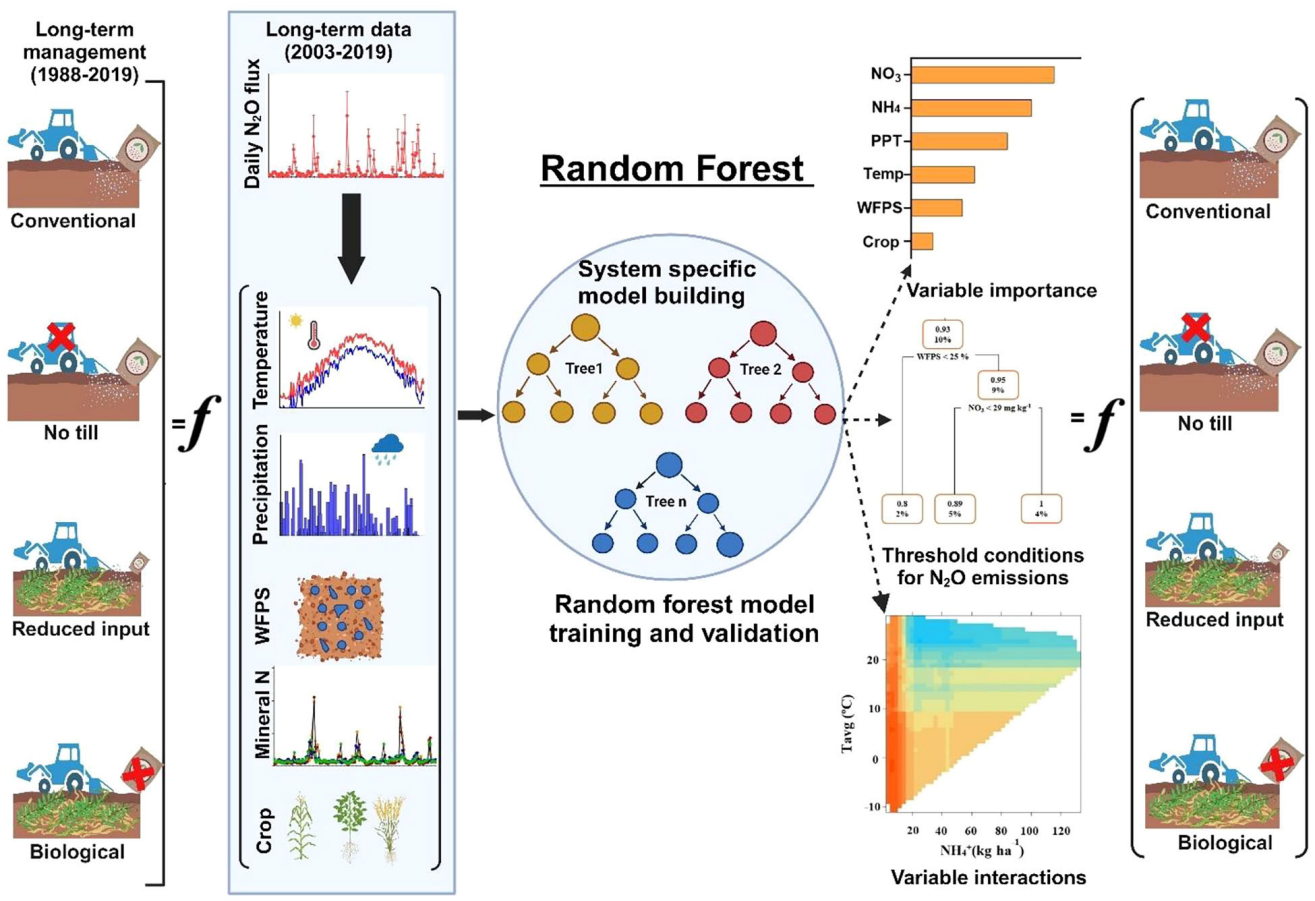
Schematic overview of random forest modeling to identify critical drivers of N_2_O emissions, their differential relationships, and threshold conditions for emissions under diverse long‐term cropping rotations.

RF and regression tree analyses were performed using the packages “*randomForest*” and “*rpart*,” respectively, in R statistical software v. 4.2.3 (R Core Team, 2023) to examine dynamic controls of N_2_O emissions and threshold response to critical drivers across diverse cropping systems (Figure 1). RF is an ensemble learning algorithm that combines numerous decision trees (*n*_tree_) and bagging (Breiman, 2001), wherein each tree is constructed using a bootstrap sample (called “in‐bag”) of dataset, and a random subset of total predictors (*m*_try_) is considered for node splits. The final RF prediction is the mean fitted response from all tree predictions. In each tree, about one‐third of data are left out and these are called out‐of‐bag (OOB) data, which are used to estimate percentage variation explained—a measure that indicates the goodness of OOB predictions explaining the target variance in the training dataset. More details on the RF algorithms can be found in Hoffman et al. (2018) and Saha, Basso, and Robinson (2021). The optimal number of predictor variables was determined using Pearson correlation analysis to remove the highly correlated variables (*r* > 0.75) to avoid overfitting (Figure S2). The final model includes daily N_2_O fluxes (log transformed values) as the response variable and average daily air temperature (*T*_avg_), cumulative precipitation in last 2 days (∑ppt_2d_), WFPS, ${\mathrm{NH}}_{4}^{+},{\mathrm{NO}}_{3}^{-}$, and crop as predictor variables (Table 2).

**TABLE 2:** Predictor variables supplied to the random forest model.

Variable	Variable category	Description	Unit
*T*_avg_	Climate	Growing season average temperature	°C
∑ppt_2d_	Climate	Growing season cumulative precipitation in last 2 days	mm
WFPS	Soil	Water‐filled pore space	%
${\mathrm{NH}}_{4}^{+}$	Soil	Soil ammonium	kg N ha^−1^
${\mathrm{NO}}_{3}^{-}$	Soil	Soil nitrate	kg N ha^−1^
Crop phase	Management	Growing main crop (corn, soybean, and wheat)	‐

Total 70% of the observations from the total observations (*n* = 3374) from each cropping system were randomly selected to construct the training set (*n* = 2362), and the remaining 30% were used for testing (*n* = 1012). The crop variable was used as a categorical variable with three levels in the data and encoded to dummy numbers to enhance the efficiency of the model algorithms. A 10‐fold cross‐validation scheme was applied to the training data to optimize the hyperparameters at *m*_try_ = 2 and *n*_tree_ = 500 using *seed* = 123 to get reproducible results. The model performance was evaluated by coefficient of determination (*r* ^2^), root mean square error (RMSE), and mean absolute error (MAE) between the observed and predicted daily N_2_O fluxes.

We used two inbuilt RF functions, variable importance metric (package “*vip*”) and PD (package “*pdp*”), and a decision tree (package “*rpart*”) to understand cropping system‐specific critical drivers, their interactions, and threshold conditions controlling N_2_O emissions. The variable importance measures the increase in model error in the OOB data in response to random permutation of input variables (Breiman, 2001). Larger error before and after permutation means greater importance of the variable and its contribution to the model's predictive accuracy. Top predictors were visualized using one‐ and two‐dimensional (2D) PD plots to identify the nature of the relationship between the predictor and response variables. Additionally, a single decision tree for each rotation was constructed using the *rpart* package in R to identify threshold conditions beyond which large changes in N_2_O flux behavior occur.

The linear mixed model using the package “*lmerTest*” from R statistical software v. 4.2.3 (R Core Team, 2023) was employed to analyze cumulative N_2_O emissions from five cycles of each rotation phase and the total cumulative emissions for the entire rotation (W–C–S) from 2004 to 2018. The N_2_O data were tested for normality using the Shapiro–Wilk test and were transformed using Box‐Cox transformations when needed. The treatments, crop phase, and their interactions were considered as fixed effects and blocks were treated as random effects. When main and interaction effects were significant at *α*  =  0.05, pairwise comparisons between treatments were performed with the estimated marginal mean function and a post hoc Tukey test using the package “*emmeans*.”

## 3 RESULTS

### 3.1 N_2_O emissions

**FIGURE 2:**
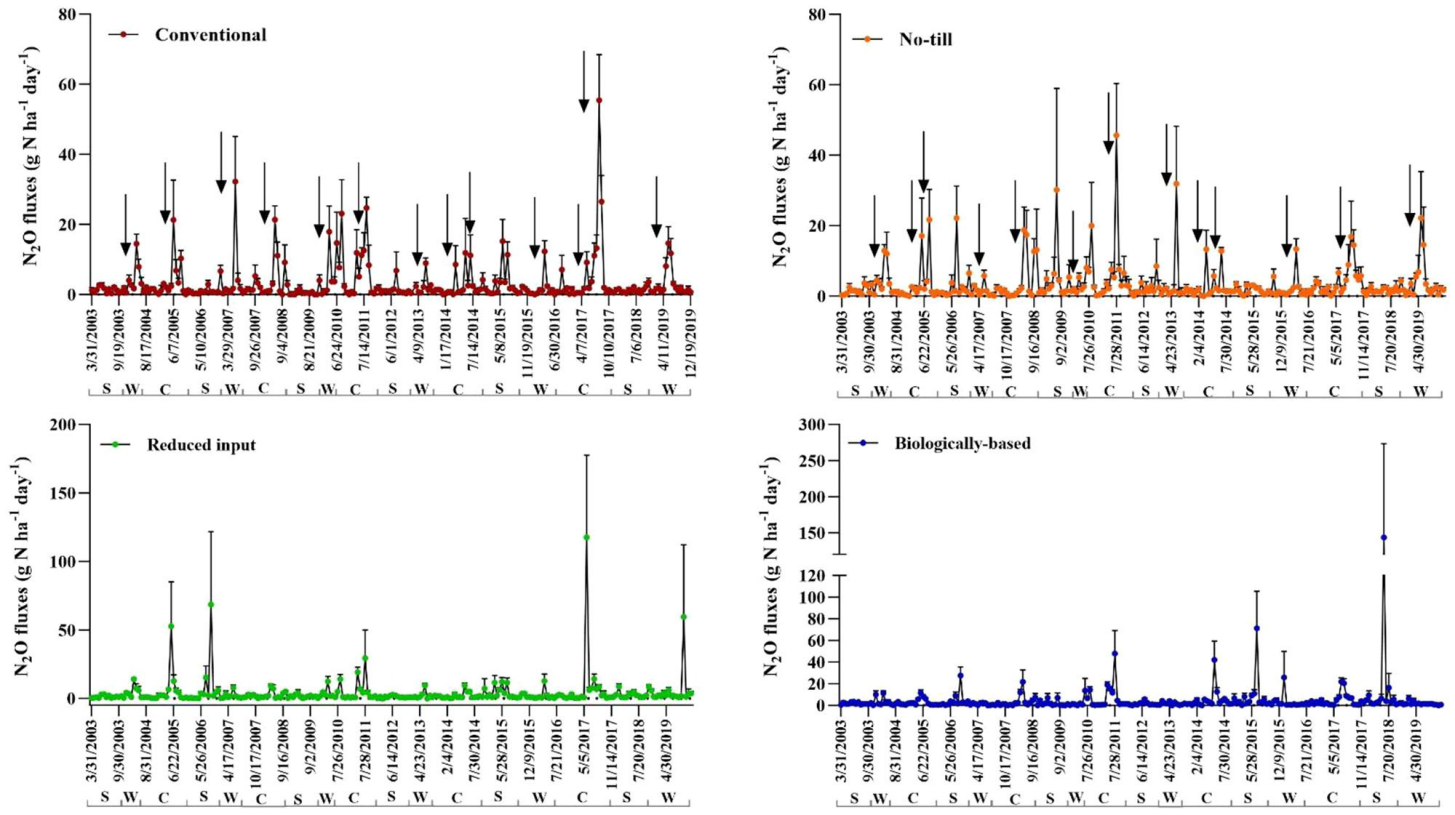
Daily N_2_O fluxes in annual systems over the period of 2003–2019. C, S, and W represent corn, soybean, and winter wheat phases, respectively. Arrows indicate times of fertilizer application in conventional and no‐till systems. Scales of the graph panels are different

**FIGURE 3:**
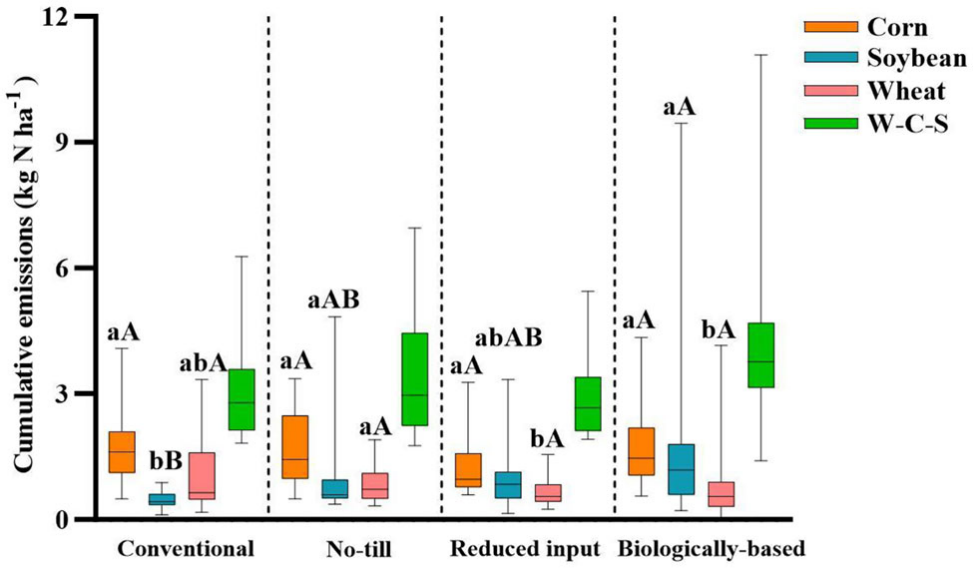
Average cumulative N_2_O emissions of five cycles of each rotation phase within each annual system and the average cumulative emissions for the entire rotation (W–C–S) from 2004 to 2018. Significant differences (*p* < 0.05) in crop phases within the same annual system are represented by different lowercase letters. Uppercase letters represent differences (*p* < 0.05) in annual systems within same crop phase. C, S, and W represent corn, soybean, and winter wheat phases, respectively.

Daily N_2_O fluxes from these annual cropping systems exhibited wide variability, ranging from −0.7 to 55.4, −0.69 to 45.6, −0.17 to 118, and −0.31 to 144 g N ha^−1^ day^−1^ in conventional, NT, reduced input, and biologically based/organic systems, respectively (Figure 2). Across five cycles of W–C–S rotations (2004–2018), total N_2_O emissions from 3‐year rotations were highest in biologically based (4.2 kg N ha^−1^), followed by NT (3.5 kg N ha^−1^), conventional (3.1 kg N ha^−1^), and reduced input systems (2.9 kg N ha^−1^), with no significant differences observed (Figure 3). In conventional system, soybean exhibited lower cumulative emissions compared to corn (*p* < 0.05), while in NT, reduced input, and biologically based systems, no discernible differences in emissions between corn and soybean were noted. Winter wheat emissions were significantly lower than emissions in corn in the reduced input system and lower than emissions in corn and soybean in the biologically based/organic system. Regardless of cropping system management, emissions from corn phase were significantly higher than emissions from soybean and wheat (1.58 vs. 1.03 vs. 0.83 kg N ha^−1^, respectively).

### 3.2 Model performance

For each annual cropping system, the RF model was trained using *T*_avg_, ∑ppt_2d_, WFPS, ${\mathrm{NH}}_{4}^{+}$, ${\mathrm{NO}}_{3}^{-}$, and crop phase as model inputs (Table 2). For the entire C–S–W rotation, the model accounted for 29%–42% of the variability between observed and predicted N_2_O fluxes, utilizing 30% of the observations for testing, which were not included in the model training (Figure S3). The highest proportion of variability in N_2_O emissions was explained by the RF model in the conventional system (42%), followed by the biologically based/organic systems (40%), the reduced input system (37%), and the NT system (29%). The RF model underpredicted N_2_O fluxes in the NT, reduced input, and biologically based systems on some occasions, with an RMSE of 0.17 in NT and biologically based systems, and 0.18 in reduced input system. The model predicted greater variability in corn phases than in soybean and wheat phases across all systems, with the highest variability predicted in biologically based (60%), followed by conventional (48%), reduced input (43%), and NT (32%) systems (Table 3). The least amount of variability was accounted for in the soybean phase in conventional (16.7%) and NT (1.46%), whereas in wheat, this was evident in reduced input (24%) and biologically based (6.1%) systems.

**TABLE 3:** Importance of variables controlling N_2_O emissions as predicted by random forest for the entire rotation (C–S–W) of each annual system and different crop phases within each system

	Conventional				No‐till				Reduced input				Biologically based			
Variable	C–S–W	C	S	W	C–S–W	C	S	W	C–S–W	C	S	W	C–S–W	C	S	W
	% increase in mean square error															
*T*_avg_	28.3 (2)	23.2	14.2	11.6	21.6 (2)	16.1	6.4	10.7	22.1 (3)	19.4	18.0	8.0	22.3 (2)	29.4	14.6	15.0
∑ppt_2d_	14.5 (4)	13.4	8.9	5.2	25.4 (1)	15.1	8.1	5.3	25.6 (2)	12.8	20.0	9.7	21.6 (3)	18.7	20.8	7.0
WFPS	13.4 (6)	9.2	10.3	5.9	8.9 (6)	10.4	2.7	2.0	13.6 (5)	9.4	9.6	4.5	20.7 (4)	25.4	12.8	3.1
${\mathrm{NO}}_{3}^{-}$	13.0 (5)	13.3	11.2	−1.9	9.9 (5)	14.3	3.7	3.6	26.2 (1)	28.9	21.5	11.1	24.1 (1)	33.3	14.5	6.5
${\mathrm{NH}}_{4}^{+}$	31.7 (1)	29.4	3.7	17.5	14.3 (3)	13.9	1.6	17.7	20.2 (4)	9.5	8.1	18.9	12.6 (6)	13.8	6.2	5.3
Crop	17.1 (3)				12.8 (4)				10.9 (6)				14.0 (5)			
	% variability explained by random forest															
	40.6	48.1	16.7	30.3	26.0	32	1.46	15.1	36.2	43.0	31.5	24.0	39.1	59.8	30.1	6.1

*Note*: The number in parentheses represents the ranking of variables in descending order for each annual system.

Abbreviations: C, corn; ${\mathrm{NH}}_{4}^{+}$, ammonium content; ${\mathrm{NO}}_{3}^{-}$, soil nitrate content; S, soybean; ∑ppt_2d_, cumulative 2‐day precipitation; *T*_avg_, average air temperature; WFPS, water‐filled pore space; W, winter wheat.

### 3.3 Critical variables for N_2_O emissions

**FIGURE 4:**
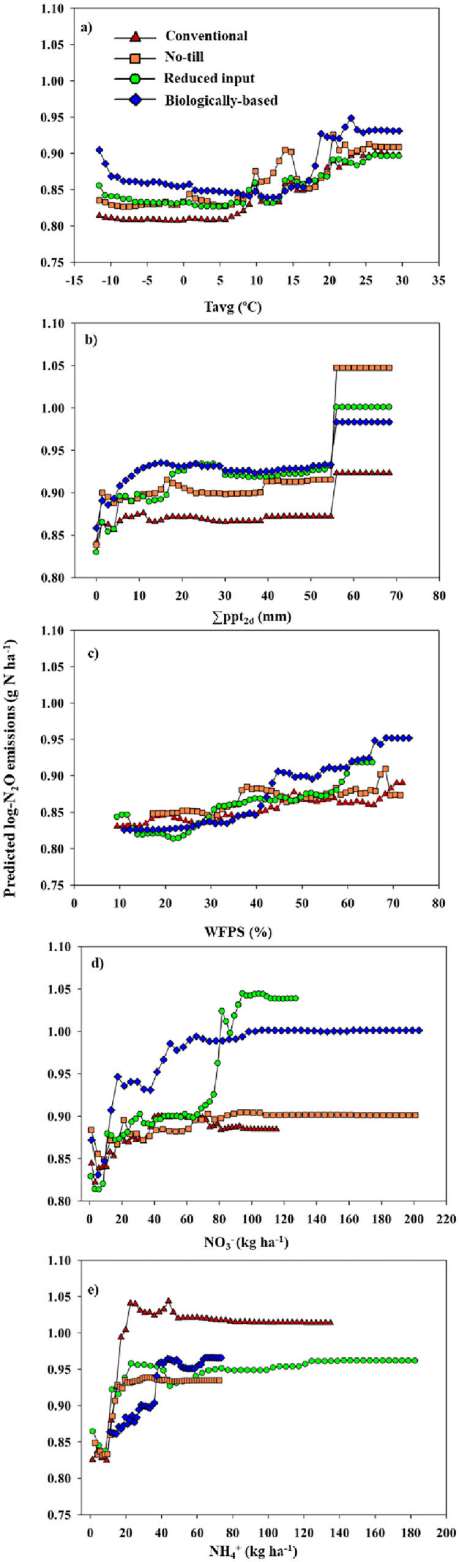
One‐dimensional partial dependence of predictor variables (a) average air temperature (*T*_avg_), (b) cumulative 2‐day precipitation (∑ppt_2d_), (c) water‐filled pore space (WFPS), (d) soil nitrate content (${\mathrm{NO}}_{3}^{-}$), and (e) soil ammonium content (${\mathrm{NH}}_{4}^{+}$) on N_2_O emissions as predicted by the random forest model under conventional, no‐till, reduced input, and biologically based/organic systems.

**FIGURE 5:**
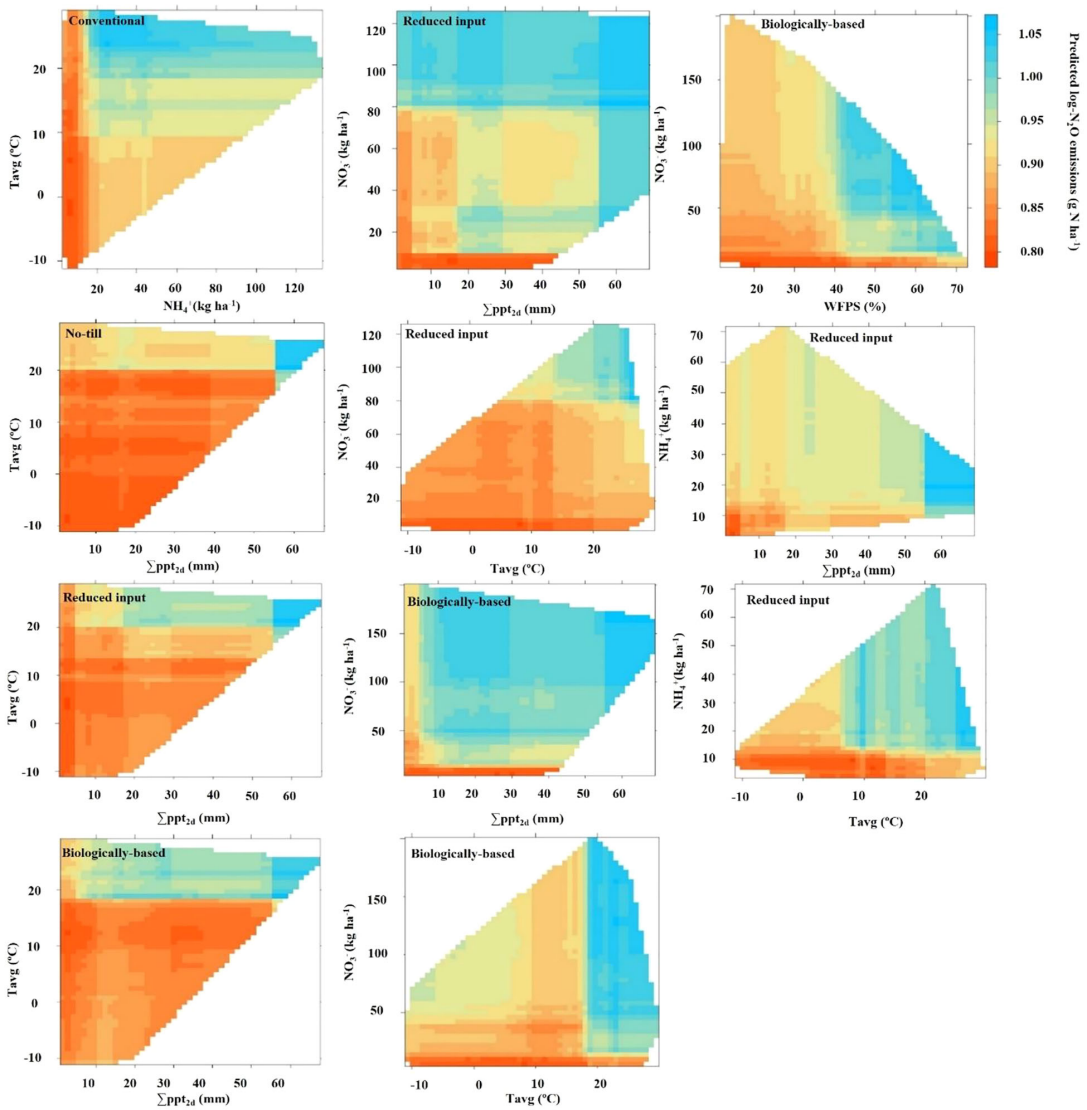
Two‐dimensional partial dependence of selected predictor variables on N_2_O emissions as predicted by the random forest model under conventional, no‐till, reduced input, and biologically based systems.

Variable importance measures identified ${\mathrm{NH}}_{4}^{+}$ and *T*_avg_ as the most influential variables in the conventional cropping system, with each variable accounting for more than 25% increase in mean square error of the OOB samples if randomly permuted (Table 3). This trend was also evident in the corn and wheat phases of the conventional system. As evident from PD plots, the model predicted high N_2_O losses when ${\mathrm{NH}}_{4}^{+}$ levels exceeded 15 kg N ha^−1^, concurrent with *T*_avg_ surpassing 20 °C (Figures 4a, 4e, and 5), which was also highlighted by the decision tree analysis (Figure S4). In the NT system, which differs from conventional only in tillage, climate variables ∑ppt_2d_ and *T*_avg_ were ranked higher than other soil and management variables (Table 3). These two variables were repeatedly used for node splitting in the decision tree (Figure S5), further highlighting their importance in controlling N_2_O fluxes from the NT system. These climate factors explained the high variability in corn phases, while ${\mathrm{NH}}_{4}^{+}$ along with *T*_avg_ emerged as primary influencers in the winter wheat phase (Table 3). The 2D plot showed high emissions when ∑ppt_2d_ reached 55 mm and *T*_avg_ exceeded 20°C (Figure 5). In the reduced input and biologically based/organic systems, where red clover precedes corn and cereal rye precedes soybean in the rotation, ${\mathrm{NO}}_{3}^{-}$ emerged as the predominant variable, with climate variables and ${\mathrm{NH}}_{4}^{+}$ following in the reduced input system, and climate variables and WFPS in the biologically based/organic system (Table 3; Figures S6 and S7). The predictability of N_2_O emissions is higher in the corn phase, with ${\mathrm{NO}}_{3}^{-}$ serving as a key predictor (Table 3), supported by the increased availability of ${\mathrm{NO}}_{3}^{-}$ resulting from the legume cover crop's being tilled into the soil before corn planting (Figure S8b). The emissions increase with an increase in ${\mathrm{NO}}_{3}^{-}$, particularly evident at approximately 10 kg N ha^−1^, with a much higher increase observed in the biologically based/organic system compared to the reduced input system (Figure 4d). A positive interaction between ${\mathrm{NO}}_{3}^{-}$ and WFPS was illustrated by the 2D plot, indicating higher N_2_O losses when WFPS exceeded ∼40% and ${\mathrm{NO}}_{3}^{-}$ levels exceeded ∼17 kg ha^−1^ (Figure 5; Figure S7).

## 4 DISCUSSION

Our findings highlight that, despite the lack of statistically significant differences in cumulative rotational N_2_O emissions for the five cycles of W–C–S rotations among annual systems, different variables emerge as the most influential factors for N_2_O fluxes in each annual system. The RF model, developed utilizing biweekly N_2_O flux manual chamber measurements from the annual cropping systems of KBS‐LTER site from 2003 to 2019, explained 29%–42% of daily flux variance in the testing dataset of the four annual systems. The climate (*T*_avg_ and ∑ppt_2d_) and soil variables (WFPS, ${\mathrm{NO}}_{3}^{-}$, and ${\mathrm{NH}}_{4}^{+}$) employed in model development are widely recognized as drivers of N_2_O emissions (Firestone & Davidson, 1989; Gelfand et al., 2016; Saha, Basso, & Robinson, 2021) and serve as easily measurable proxies for soil biophysical and biogeochemical processes. The RF model has been extensively validated and demonstrated its reliability in predicting N_2_O emissions in croplands, with *r* ^2^ values ranging from 0.38 to 0.73 (Glenn et al., 2021; Joshi et al., 2024; Liao et al., 2023; Philibert et al., 2013; Saha, Basso, & Robinson, 2021; Yin et al., 2022).

In the conventional cropping system, soil ${\mathrm{NH}}_{4}^{+}$ and *T*_avg_ emerged as particularly significant predictors for N_2_O fluxes. The model predicted a higher risk of N_2_O emissions following N fertilization application (${\mathrm{NH}}_{4}^{+}$ > 15 kg N ha⁻¹) during periods of high air temperatures (*T*_avg_ > 20°C) (Figure 5; Figure S4). Emissions typically peak following the side‐dress application of UAN during the corn and wheat phases, coinciding with high air temperatures (Figure 2), also reported in many other studies in temperate climates (Adviento‐Borbe et al., 2007; Gasche & Papen, 2002; Kitzler et al., 2006; Ma et al., 2010). This observation might elucidate the relatively higher predictability for fluxes modeled during the corn (48%) and winter wheat (32%) phases compared to the soybean phase (16.7%) (Table 3), with ${\mathrm{NH}}_{4}^{+}$ and *T*_avg_ emerging as the topmost variables within the corn and wheat phases. Microbial activities during nitrification and denitrification tend to be more active under higher temperatures (Kätterer et al., 1998), suggesting that air temperature plays an important role in regulating the rate of N_2_O emissions (Rashti et al., 2015). Our findings, which highlight the primary influence of ${\mathrm{NH}}_{4}^{+}$ and *T*_avg_ in conventional system, suggest that the emissions might be linked to strong nitrification activity in this system. High ${\mathrm{NH}}_{4}^{+}$ levels have elsewhere also been associated with elevated N_2_O emissions (Breitenbeck et al., 1980; Peyrard et al., 2016). However, Liang and Robertson (2021) conclude that nitrification is a minor source of N_2_O emissions in this system based on combining soil‐specific kinetics of nitrification‐derived N_2_O with 25 years of N_2_O flux measurements. In that study, the maximum potential contributions from nitrification to in situ N_2_O fluxes were found to be 13%–17%, with actual contributions likely only 1%–2%. Nitrification is rapid in these soils (Millar & Robertson, 2015), such that high ${\mathrm{NH}}_{4}^{+}$ levels can simultaneously indicate high ${\mathrm{NO}}_{3}^{-}$ availability despite lower soil ${\mathrm{NO}}_{3}^{-}$ levels if ${\mathrm{NO}}_{3}^{-}$ pools are rapidly depleted by plant uptake, denitrification, or leaching (Gelfand et al., 2016; Syswerda et al., 2012). Inorganic N availability might be better (less ambiguously) assessed as a driver of N_2_O fluxes by combining ${\mathrm{NH}}_{4}^{+}$ and ${\mathrm{NO}}_{3}^{-}$ into a single soil mineral N predictor.

In the NT system, where all other management aspects remain identical to the conventional system except for the adoption of NT practices, the RF model had the least predictability (29%), reflecting the complexity of the underlying processes and drivers of N_2_O production in NT systems. Despite emissions’ rising similarly to those in the conventional system after fertilizer application (Figure 2), climate variables took precedence over the effects of soil mineral N variability. Changing climate factors can regulate soil O_2_ dynamics, serve as proxies for soil biophysical processes, and impact N_2_O emissions (Song et al., 2019). Precipitation primarily changes soil O_2_ concentrations by displacing soil air with water and serves as a reliable indicator of soil redox potential, affecting conditions that govern soil mineral N transformations and leading to N_2_O production (Linn & Doran, 1984; Rochette et al., 2018). High *T*_avg_ (>∼20°C) coupled with high ∑ppt_2d_ (∼55 mm) (Figure 5) can simultaneously promote microbial O_2_ consumption via enhanced microbial activity and inhibited O_2_ diffusion. Rochette et al. (2018) in their study on soil N_2_O emissions and their controls in temperate climates of Canada reported that precipitation plays a primary role in determining N_2_O emissions, and that environmental conditions can mask the impact of soil N content under well‐aerated conditions. Grandy et al. (2006) documented increased aggregation and enhanced soil structure in the same NT system described here. Microsites or pores within these stable aggregates under long‐term NT can lead to low and varying O_2_ levels across the aggregate radius (Sexstone et al., 1985; Song et al., 2019), a dynamic not captured by WFPS, which typically serves as a proxy for soil O_2_ fluctuation influenced by soil moisture levels (Dobbie & Smith, 2001, 2003). Likewise, differences in soil pores in well‐structured soils can lead to microsite differences in water and O_2_ levels that can drive differences in N_2_O production (Kravchenko et al., 2017). Such heterogeneity in O_2_ under well‐structured soils could strongly impact N transformations and N_2_O production. Thus, soil pore structure and soil O_2_ parameters, which may serve as better proxies than WFPS and reliable predictors of N_2_O emissions, could enhance the predictive capability of the RF model in the NT system. However, we acknowledge the challenges associated with accurately capturing high‐resolution soil O_2_ consumption within pore spaces. Furthermore, the availability of data on SOC may provide additional predictive capacity for the model. This is particularly significant, as NT is proposed as one of the main measures to reduce N_2_O emissions and increase C sequestration (Van Kessel et al., 2013). A better understanding of controls of N_2_O emissions in NT soils is required.

In contrast to the conventional and NT systems, the cover‐cropped reduced input and biologically based/organic systems revealed soil ${\mathrm{NO}}_{3}^{-}$ as the primary variable explaining the largest portion of the variation in N_2_O emissions (24%–26%; Table 3). This is particularly evident after incorporating red clover before corn planting, where there is a notable increase in ${\mathrm{NO}}_{3}^{-}$ availability (Figure S8b) and, concomitantly, in emissions (Figure 2). The model's high predictability of N_2_O fluxes in the corn phase (43% in the reduced input and 60% in the biologically based/organic system), with ${\mathrm{NO}}_{3}^{-}$ as the top variable, further demonstrates this. Furthermore, the lower predictive value observed for wheat in these systems, along with the lower ranking of ${\mathrm{NO}}_{3}^{-}$ within the wheat system, suggests that there is a limited carryover effect of the decomposition of leguminous cover crop biomass into the wheat phase. Increased N_2_O associated with legume crops could be attributed to enhanced N release from decomposing leguminous residues (Abalos et al., 2022; Rochette & Janzen, 2005). In these systems, chisel tillage may enhance N mineralization by incorporating legume cover crops into the soil when temperatures are sufficiently warm to support active decomposition. This is further supported by the prediction of an increase in emissions with rising air temperature (Figure 4a). The simultaneous availability of easily degraded N and C from organic inputs increases the risk of high N_2_O emissions by enhancing biological activity, leading to soil O_2_ depletion through enhanced soil respiration and increased denitrification (Hansen et al., 2019; Lussich et al., 2024).

The heightened risk of significant N_2_O emissions following precipitation events when WFPS exceeds 53% and ${\mathrm{NO}}_{3}^{-}$ availability surpasses 17 kg ha^−1^ (Figure 5) indicates N_2_O likely originated from denitrification. This threshold value for WFPS aligns with the findings of Peyrard et al. (2016), although when denitrification is involved, the WFPS threshold is often higher, ranging from 60% to 80% (Davidson, 1991). The wetness‐independent anoxia created by decomposing legume residues might partly explain N_2_O production, a phenomenon not captured by WFPS. Respiration‐induced anoxia caused by decomposing cover crop residues can promote N_2_O emissions, even under suboptimal WFPS (50%) conditions for denitrification (Lussich et al., 2024). This could also hold true for the reduced input system, where the predictive value of WFPS is lower and no interaction of ${\mathrm{NO}}_{3}^{-}$ with ∑ppt_2d_ was observed. Future advancements in our understanding and data availability regarding the response of N_2_O to soil O_2_ consumption during the decomposition of cover crop residues may enhance the predictive capacity of models in cover crop‐based cropping systems.

## 5 CONCLUSIONS

Results underscore the efficacy of a decision tree‐based nonlinear machine learning model for identifying key variables, their threshold conditions, and complex interactions in influencing N_2_O emissions in intensively managed annual cropping systems. Our findings leveraging long‐term data reveal that differential controls of N_2_O emissions are important under different cropping system managements. In the conventional system, soil ammonium and air temperature emerged as the primary influencers of N_2_O emissions, while in the NT system, climatic conditions—particularly precipitation and air temperature—exerted the greatest impact on emissions. Nitrate availability from legume cover crops drove N_2_O emissions in the reduced input and biologically based/organic systems.

Although our RF model effectively predicted 29%–42% of the daily variability in N_2_O fluxes from intensively managed cropping systems, the model can be further improved by incorporating long‐term high‐frequency observations from automated flux chambers and by including soil organic carbon data, as NT and cover crop systems can influence N_2_O emissions by enhancing soil carbon content. Considering the challenge posed by the generalizability of the RF model, its application to other regions and crops necessitates further enhancements in model training based on diverse data sources encompassing various soils, climates, crops, and management conditions.

## AUTHOR CONTRIBUTIONS

**Jashanjeet Kaur Dhaliwal**: Conceptualization; formal analysis; investigation; methodology; software; validation; visualization; writing—original draft; writing—review and editing. **Dinesh Panday**: Data curation; formal analysis; methodology; visualization; writing—original draft; writing—review and editing. **G. Philip Robertson**: Data curation; funding acquisition; methodology; resources; supervision; writing—review and editing. **Debasish Saha**: Conceptualization; formal analysis; funding acquisition; investigation; methodology; project administration; resources; supervision; writing—original draft; writing—review and editing.

## CONFLICT OF INTEREST STATEMENT

The authors declare no conflicts of interest.

## Supporting information




**Figure S1** Maximum and minimum air temperature and precipitation distribution during the crop growing period (2003 to 2019): a) annual average maximum and minimum temperature and cumulative precipitation and b) monthly average maximum and minimum temperature and cumulative precipitation
**Figure S2** Pearson's correlation matrix among all measured variables under four annual systems. The color and shade of the squares denote the direction and magnitude of the relationship and ‘×’ represents *p* >0.05 between two variables. N_2_O; nitrous oxide, NO₃⁻; soil nitrate content, NH₄⁺; soil ammonium content, WFPS; water‐filled pore space, ∑ppt_2d_; cumulative 2‐day precipitation, Tavg; average air temperature
**Figure S3** Observed vs random forest predicted N_2_O fluxes under a) Conventional, b) No‐till, c) Reduced input, and d) Biologically‐based/organic cropping systems. The solid lines indicate a 1:1 relation between the observation and predicted N_2_O fluxes for training and testing data. r^2^ = coefficient of determination, RMSE = root mean square error, and MAE = mean absolute error
**Figure S4** Decision tree for predicting N_2_O emissions using training data in Conventional system based on Tavg; average air temperature, ∑ppt_2d_; cumulative 2‐day precipitation, WFPS; water‐filled pore space, NO₃⁻; soil nitrate content, and NH₄⁺; soil ammonium content
**Figure S5** Decision tree for predicting N_2_O emissions using training data in No‐till system based on Tavg; average air temperature, ∑ppt_2d_; cumulative 2‐day precipitation, WFPS; water‐filled pore space, NO₃⁻; soil nitrate content, and NH₄⁺; soil ammonium content
**Figure S6** Decision tree for predicting N_2_O emissions using training data in Reduced input system based on Tavg; average air temperature, ∑ppt_2d_; cumulative 2‐day precipitation, WFPS; water‐filled pore space, NO₃⁻; soil nitrate content, and NH₄⁺; soil ammonium content
**Figure S7** Decision tree for predicting N_2_O emissions using training data in Biologically‐based/organic system based on Tavg; average air temperature, ∑ppt_2d_; cumulative 2‐day precipitation, WFPS; water‐filled pore space, NO₃⁻; soil nitrate content, and NH₄⁺; soil ammonium content
**Figure S8** Soil N availability‐ NH₄⁺ (a) and NO₃⁻ (b) over the period of 2003‐2019 under four annual systems studied. S = soybean, W = winter wheat, and C = corn phases. *Note: Figures are prepared using interpolation data to align them with daily N_2_O flux data
**Table S1** Average daily N_2_O emissions (mean ± standard error) from annual cropping systems.

## Data Availability

Data supporting the findings and R codes used in this study are openly available in Dryad at https://doi.org/10.5061/dryad.9cnp5hqv1.
