# Supplementary material for: Machine learning reveals dynamic controls of soil nitrous oxide emissions from diverse long‐term cropping systems
Source: J Environ Qual. 2024 Oct 9;54(1):132–46. doi: 10.1002/jeq2.20637 (PMC11718152; doi:10.1002/jeq2.20637)
Supplement: Supplementary file 1 — Figure S1 Maximum and minimum air temperature and precipitation distribution during the crop growing period (2003 to 2019): a) annual average maximum and minimum temperature and cumulative precipitation and b) monthly average maximum and minimum temperature and cumulative precipitation Figure S2 Pearson's correlation matrix among all measured variables under four annual systems. The color and shade of the squares denote the direction and magnitude of the relationship and ‘×’ represents p >0.05 between two variables. N2O; nitrous oxide, NO₃⁻; soil nitrate content, NH₄⁺; soil ammonium content, WFPS; water‐filled pore space, ∑ppt2d; cumulative 2‐day precipitation, Tavg; average air temperature Figure S3 Observed vs random forest predicted N2O fluxes under a) Conventional, b) No‐till, c) Reduced input, and d) Biologically‐based/organic cropping systems. The solid lines indicate a 1:1 relation between the observation and predicted N2O fluxes for training and testing data. r2 = coefficient of determination, RMSE = root mean square error, and MAE = mean absolute error Figure S4 Decision tree for predicting N2O emissions using training data in Conventional system based on Tavg; average air temperature, ∑ppt2d; cumulative 2‐day precipitation, WFPS; water‐filled pore space, NO₃⁻; soil nitrate content, and NH₄⁺; soil ammonium content Figure S5 Decision tree for predicting N2O emissions using training data in No‐till system based on Tavg; average air temperature, ∑ppt2d; cumulative 2‐day precipitation, WFPS; water‐filled pore space, NO₃⁻; soil nitrate content, and NH₄⁺; soil ammonium content Figure S6 Decision tree for predicting N2O emissions using training data in Reduced input system based on Tavg; average air temperature, ∑ppt2d; cumulative 2‐day precipitation, WFPS; water‐filled pore space, NO₃⁻; soil nitrate content, and NH₄⁺; soil ammonium content Figure S7 Decision tree for predicting N2O emissions using training data in Biologically‐based/organic system based on Tavg; [file JEQ2-54-132-s001.docx]

**Supplemental Data**

**Machine Learning Reveals Dynamic Controls of Soil Nitrous Oxide (N_2_O) Emissions from Diverse Long-term Cropping Systems**

^1^Jashanjeet Kaur Dhaliwal, ^1, 2^Dinesh Panday, ^3,4^G. Philip Robertson, ^1^Debasish Saha*

***^1^****Biosystems Engineering and Soil Science, University of Tennessee – Knoxville, Tennessee, USA*

***^2^****Rodale Institute, Kutztown, Pennsylvania, USA*

*^3^W. K. Kellogg Biological Station, Michigan State University, Hickory Corners, Michigan, USA*

*^4^Department of Plant, Soil, and Microbial Sciences, Michigan State University, East Lansing, Michigan USA*

*^*^Corresponding author (dsaha3@utk.edu)*


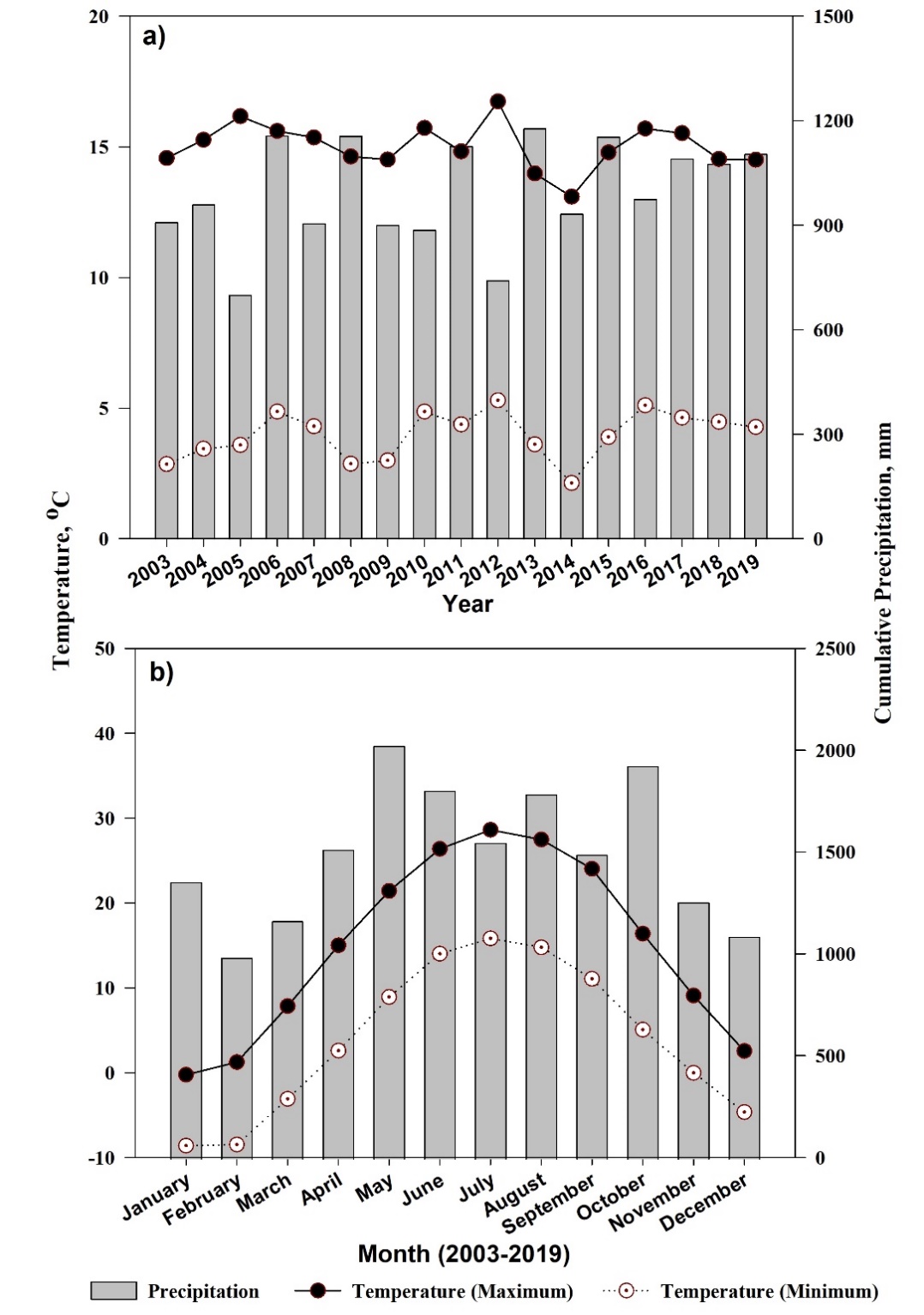


**Figure S1** Maximum and minimum air temperature and precipitation distribution during the crop growing period (2003 to 2019): a) annual average maximum and minimum temperature and cumulative precipitation and b) monthly average maximum and minimum temperature and cumulative precipitation


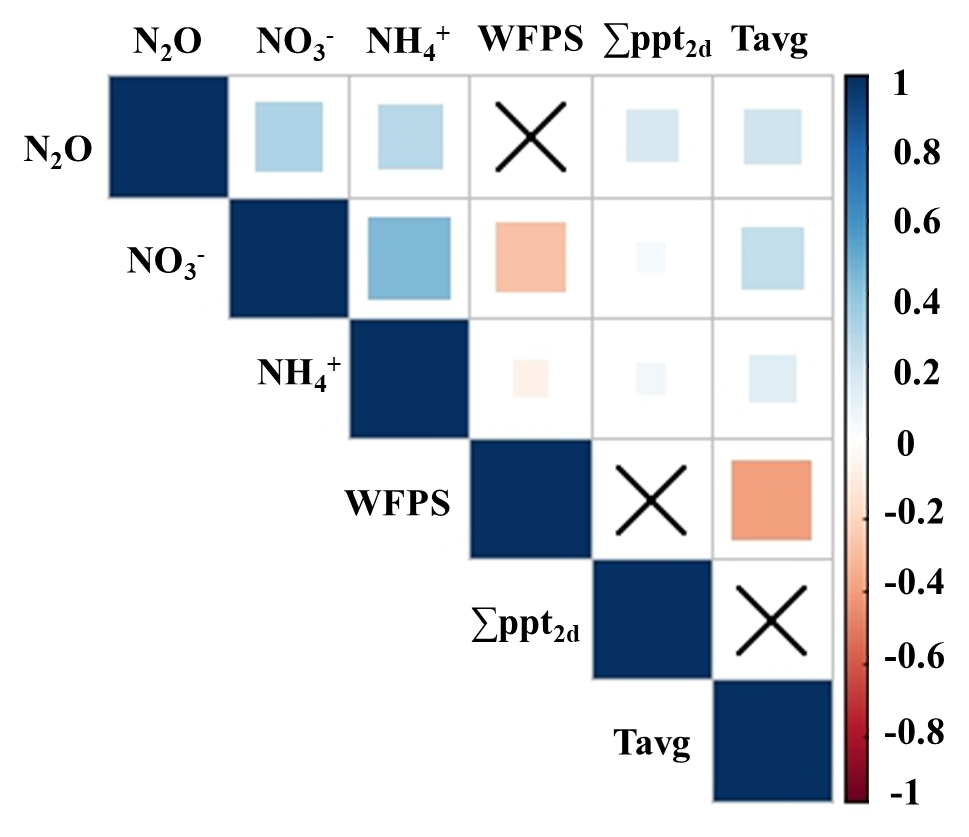


**Figure S2** Pearson’s correlation matrix among all measured variables under four annual systems. The color and shade of the squares denote the direction and magnitude of the relationship and ‘×’ represents *p* >0.05 between two variables. N_2_O; nitrous oxide, NO₃⁻; soil nitrate content, NH₄⁺; soil ammonium content, WFPS; water-filled pore space, ∑ppt_2d_; cumulative 2-day precipitation, Tavg; average air temperature


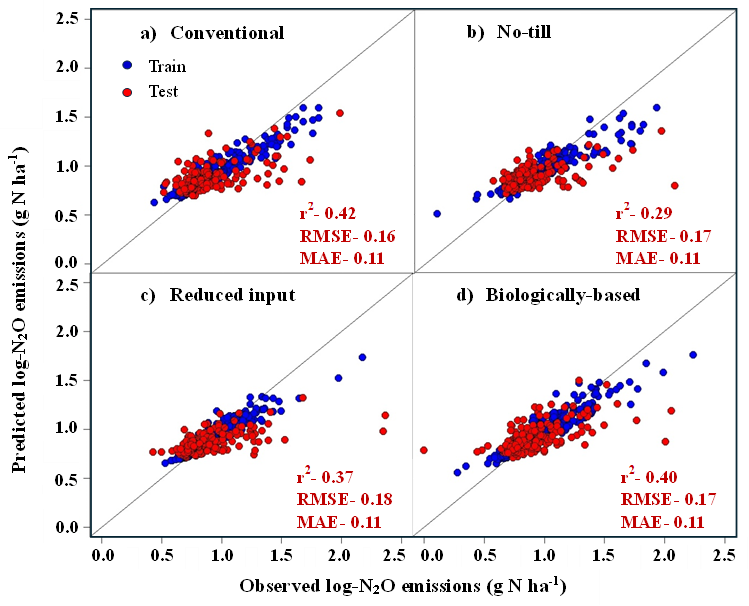


**Figure S3** Observed vs random forest predicted N_2_O fluxes under a) Conventional, b) No-till, c) Reduced input, and d) Biologically-based/organic cropping systems. The solid lines indicate a 1:1 relation between the observation and predicted N_2_O fluxes for training and testing data. r^2^ = coefficient of determination, RMSE = root mean square error, and MAE = mean absolute error


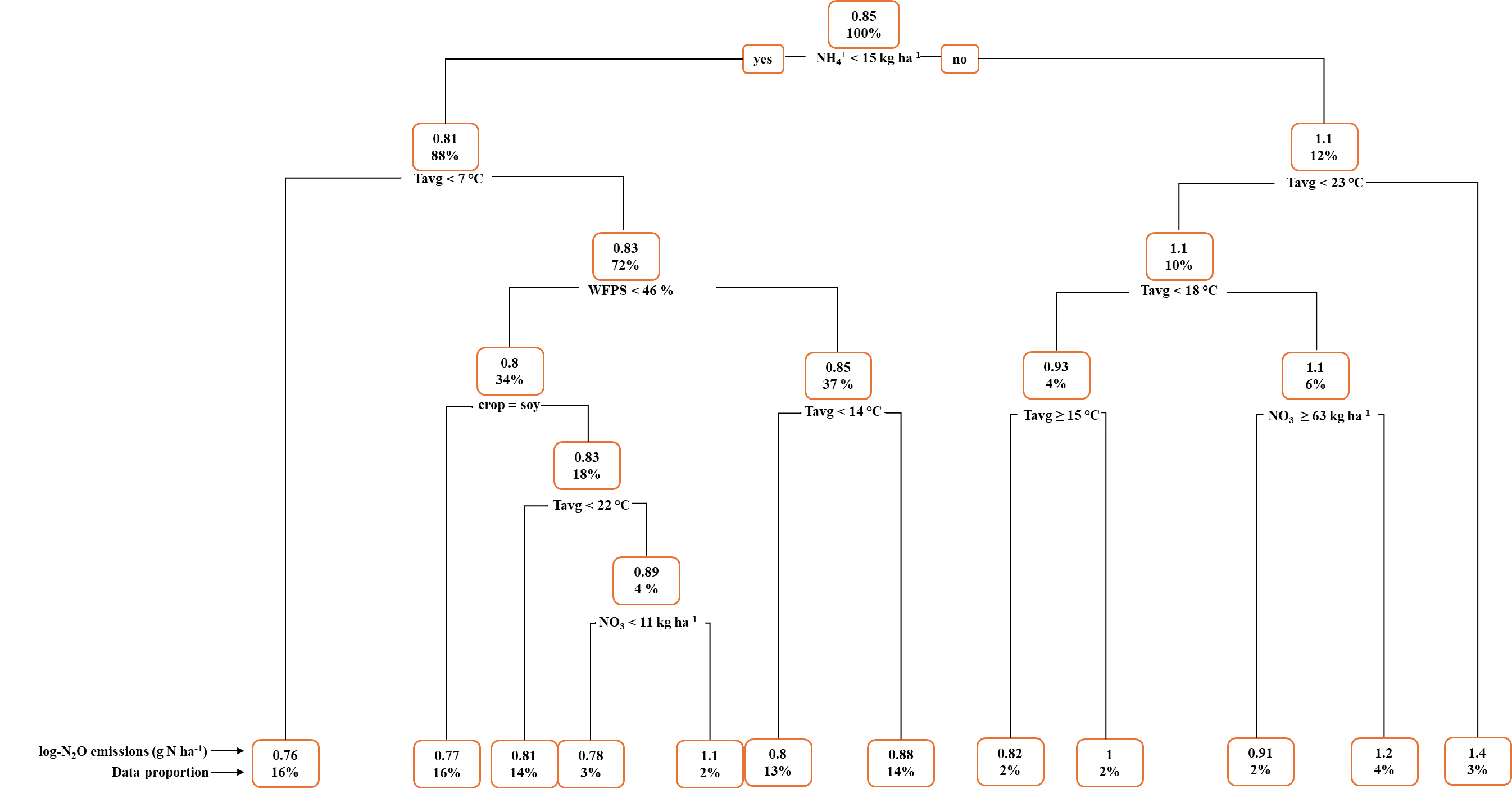


**Figure S4** Decision tree for predicting N_2_O emissions using training data in Conventional system based on Tavg; average air temperature, ∑ppt_2d_; cumulative 2-day precipitation, WFPS; water-filled pore space, NO₃⁻; soil nitrate content, and NH₄⁺; soil ammonium content


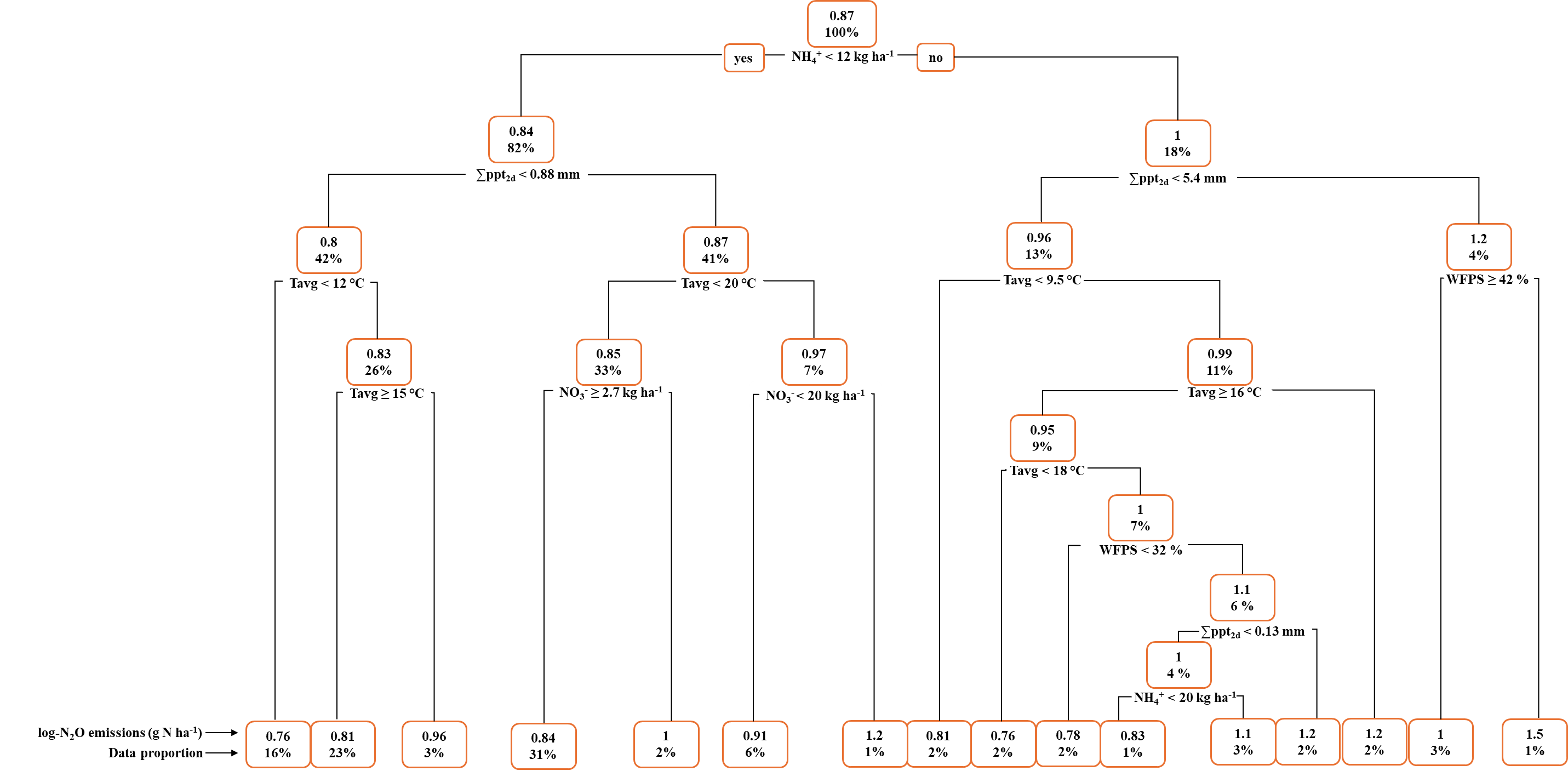


**Figure S5** Decision tree for predicting N_2_O emissions using training data in No-till system based on Tavg; average air temperature, ∑ppt_2d_; cumulative 2-day precipitation, WFPS; water-filled pore space, NO₃⁻; soil nitrate content, and NH₄⁺; soil ammonium content


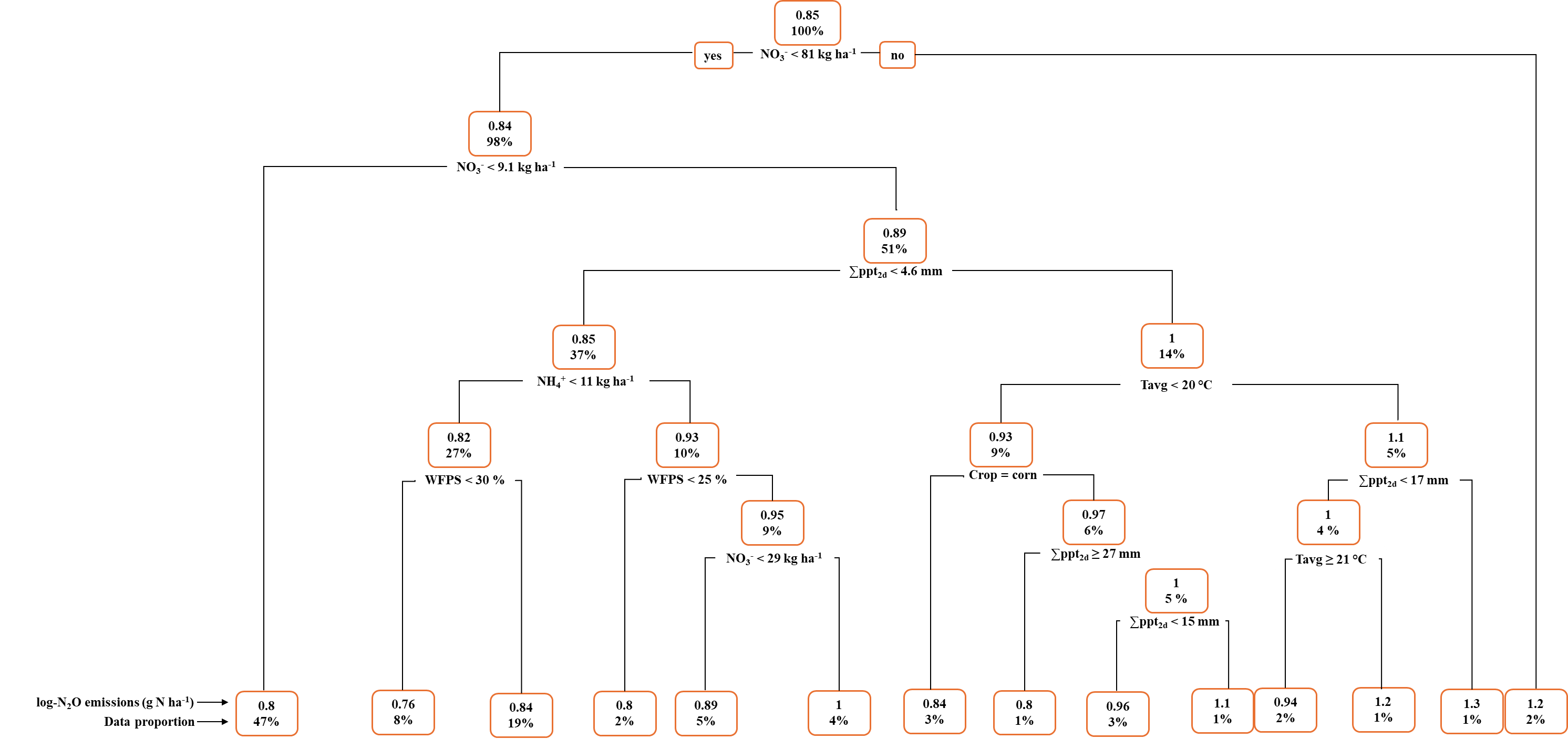


**Figure S6** Decision tree for predicting N_2_O emissions using training data in Reduced input system based on Tavg; average air temperature, ∑ppt_2d_; cumulative 2-day precipitation, WFPS; water-filled pore space, NO₃⁻; soil nitrate content, and NH₄⁺; soil ammonium content


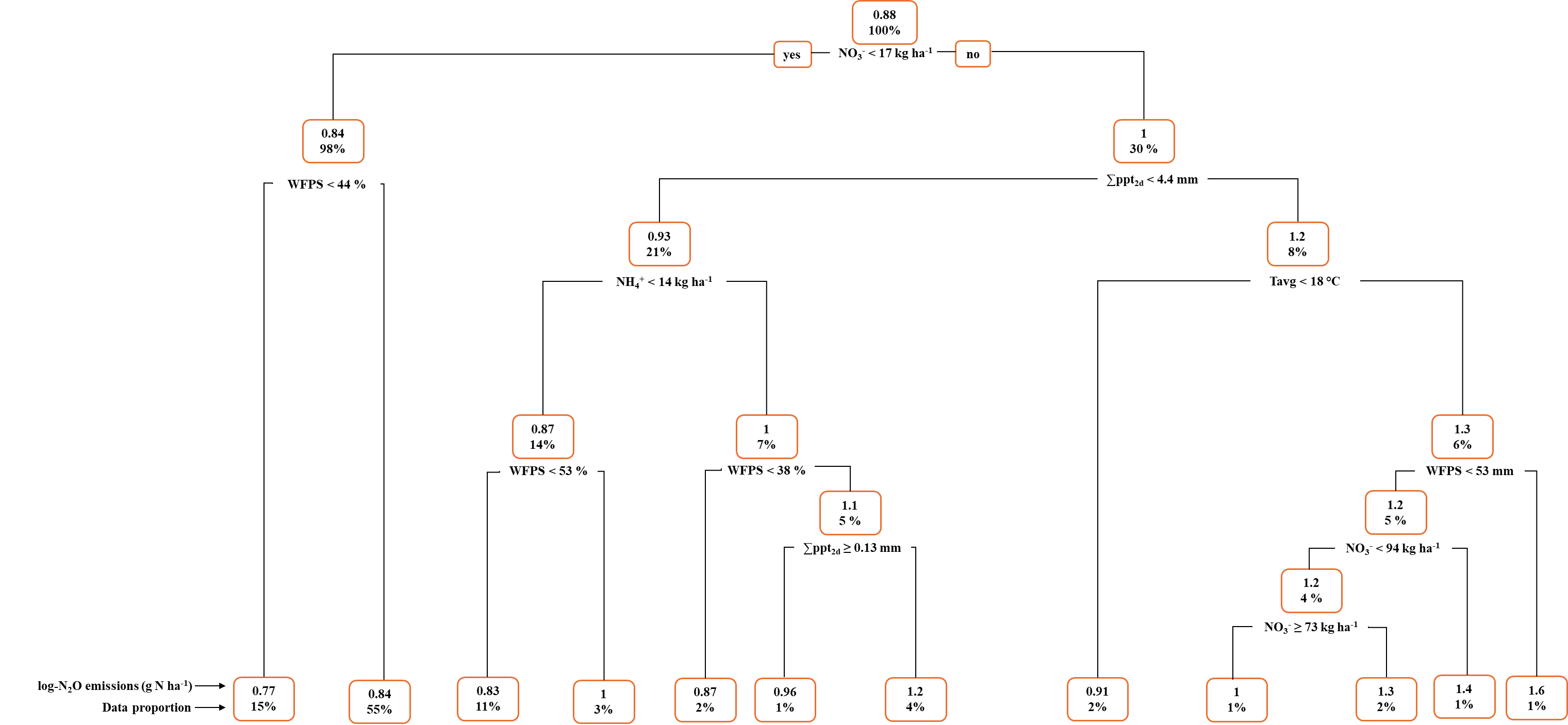


**Figure S7** Decision tree for predicting N_2_O emissions using training data in Biologically-based/organic system based on Tavg; average air temperature, ∑ppt_2d_; cumulative 2-day precipitation, WFPS; water-filled pore space, , NO₃⁻; soil nitrate content, and NH₄⁺; soil ammonium content

**
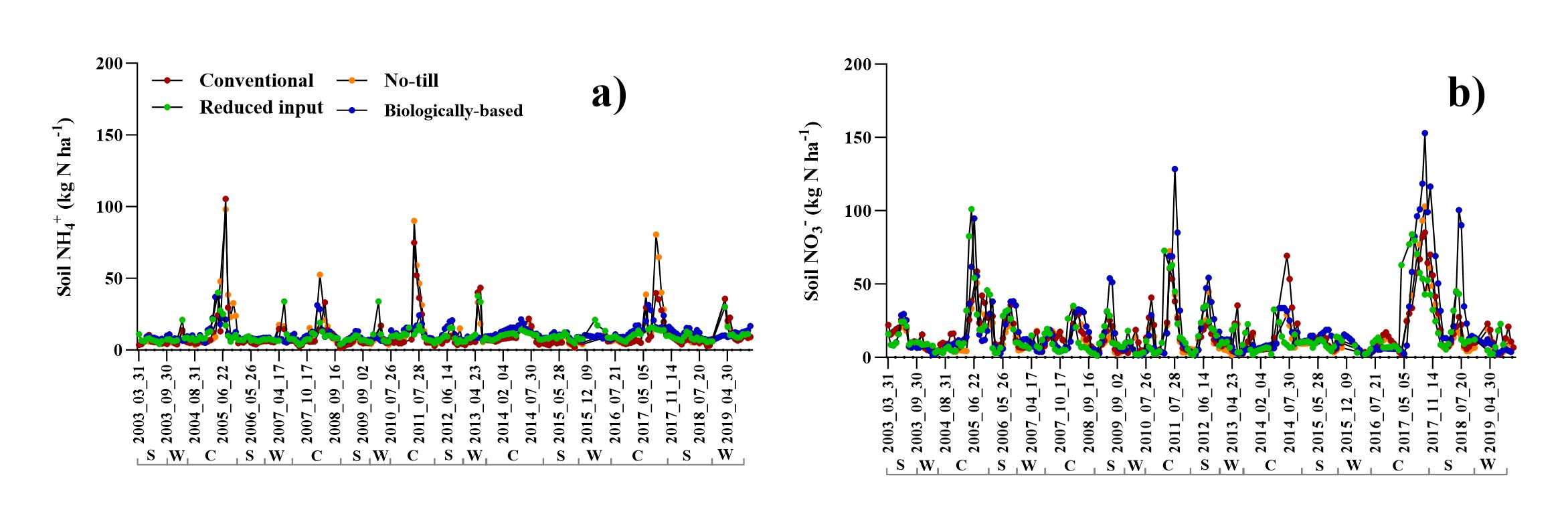
Figure S8** Soil N availability- NH₄⁺ (a) and NO₃⁻ (b) over the period of 2003-2019 under four annual systems studied. S = soybean, W = winter wheat, and C = corn phases.

*Note: Figures are prepared using interpolation data to align them with daily N_2_O flux data

**Table S1 Average daily N_2_O emissions (mean ± standard error) from annual cropping systems**

| Treatments | Average daily N_2_O fluxes (g N ha^-1^ day^-1^) | | | | | |
| --- | --- | --- | --- | --- | --- | --- |
|  | Conventional | No-till | Reduced input | Biologically-based | | Mean |
| Corn | 4.61 (±0.54) aAB | 4.30 (±0.51) aAB | 3.46 (±0.54) aB | 4.74 (± 0.50) aA | 4.28 | |
| Soybean | 1.58 (±0.18) bC | 2.78 (±0.48) aBC | 3.34 (±0.75) aAB | 6.15 (±1.89) aA | 3.46 | |
| Wheat | 4.23 (±0.61) aA | 4.15 (±0.69) aA | 4.00 (±1.15) aAB | 2.43 (±0.45) bB | 3.70 | |
| Mean | 3.47 | 3.74 | 3.59 | 4.44 |  | |

Significant differences (*P < 0.05*) in crops within the same annual system are represented by different lowercase letters. Uppercase letters represent differences (*P < 0.05*) in annual systems within same crop phase.
